# Articulating Artificial Dentures*Extracted from a paper published in “The National Dental Journal” for April, 1919.

**Published:** 1919-06

**Authors:** O. A. Weiss


### ARTICULATING ARTIFICIAL DENTURES *

BY O. A. WEISS, D.D.S.

In the College of Dentistry of the University of Minnesota we use the essayists' modification of the Bonwill articulator in which we have a fixed condyloid path of an inclination of 35 degrees. When the teeth have been articulated in the articulator they are always tried in the mouth for correction. It is my belief that until we have an articulator that reproduces exactly the movements of the mandible in every case without an extensive and difficult technic, the only safe way is to test and perfect the articulation in the mouth. We feel that our method is comparatively simple and readily within the reach of dental students, and at the same time sufficiently accurate in its principal features to meet the demands; in other words, our method is simple, efficient and practicable.

Our students make from eight to ten dentures each and with classes numbering from eighty to one hundred members, it affords us ample opportunity for observation. While we do not claim to satisfy every patient, yet the number of dissatisfied ones is exceedingly small.

In addition to a modified articulator which enables us to carry out the principles of articulation enunciated by Dr. Bonwill, we have made two other important modifications of Bonwill's teachings; namely, we make as little overbite as possible, and we set the teeth as nearly over the center of the ridges as is permissible considering, of course, facial restoration as affected by the anterior teeth.

Dr. Bonwill used an overbite of one-eighth of an inch or even more. While articulation can be made with such an overbite, I consider this impracticable—a point I emphasized in a paper before the Minnesota State Dental

* Extracted from a paper published in "The National Dental Journal" for April, 1919.

Association in 1902, and which has likewise been emphasized by such an eminent investigator as Dr. Gysi—because it necessitates long cusps in the bicuspids and molars which results in such deep meshing of the cusps as to lock them and prevent lateral movement of the teeth. Any attempt at lateral movement results in movement of the mucous membrane and underlying tissue upon which the plates rest, or the mandible slides from under the plate except in cases where there is a very prominent alveolar ridge. In testing such articulation it was almost always observed that the teeth remained locked in attempting lateral movement, instead of gliding over each other as expected in the grinding action. Only when the patient is instructed to occlude very lightly in order to permit the teeth to glide over each other is it possible to get lateral movement of the teeth.

With a very short overbite of the incisors the occlusal surfaces of the bicuspids and molars will be comparatively flat, thus readily permitting lateral movement as required in articulation.

That long cusps with consequent deep meshing would be more efficient as organs of mastication may readily be admitted, provided the teeth are securely anchored to the jaws, but with such teeth mounted upon plates, the lower of which is usually held in position with very little more force than its own weight it must have been often observed by experienced and careful investigators that such teeth remained in mesh or locked when attempting lateral movement as previously explained.

Teeth with deeply meshing cusps are used, however, and successfully, but it remains to be demonstrated that they are used in any other movement than occlusion alone, and they are not used in articulation or lateral movement as can be done with the natural teeth.

It is here where comparative tests may shed some light. It is quite possible, even probable, that deeply meshing cusps may be more efficient organs of mastication in artificial dentures, even though lateral movement or articulation is not permissible, than flat cusps which would permit lateral movement.

The relation of the teeth to the alveolar ridge is also an important matter. Dr. Bonwill claimed it made no difference what the relation of the teeth to the alveolar ridge might be, provided the teeth articulate properly and that the arch line ran straight back from the cuspids to the condyles. This involves a question of leverage pure and simple. If the teeth are set buccally or labially from the center of the alveolar ridge, leverage is produced in mastication which tends to displacement of the plates. Tendency to displacement or the amount of leverage depends directly upon the distance the teeth are set outside of the center of the alveolar ridge.

After the teeth have been articulated in the articulator they are tried in the mouth. First, of course, the occlusion is tested; we must prove that all of the teeth are in occlusion with the mouth closed in normal repose. For this, various tests are used similar to those employed in taking the bite, for which one of the best is to have the patient place the tip of the tongue far back upon the palate; this alone, however, should not be relied upon. When the correct relation of the jaws has been found we then test the occlusion, not merely by looking at it, but by the use of a thin stiff spatula forced lightly between the occluding teeth and then exerting a rotary action upon the spatula by which we will have opposing force applied to the upper and lower teeth. Usually the teeth will be found in occlusal contact when looking at them, but by the use of a spatula as just described, we will frequently find that the teeth are forced apart indicating that one or possibly both plates were not resting upon the ridges. When the bite has been correctly taken and the casts properly mounted in the articulator the occlusion will always test correct when trying the plates in the mouth. Should the occlusion be found faulty, it, of course, must be corrected; this will depend entirely upon the character of the error. Usually the error will be that the teeth are too long or too short upon one side. This is quite easily remedied. If there is error in the labio-lingual or bucco-lingual relation of the teeth it may be necessary to take the bite anew and articulate the teeth again.

After the occlusion has been tested the lateral movements and the protruding or incisive movement are tested in similar manner.

In testing the protruding or incisive movement we must remember that the curvature in the occlusal line—the compensating curve—must be in agreement with the inclination of the condyloid path and the overbite. If in protruding the mandible we should find that there is incisal contact only it signifies that there is insufficient curvature in the occlusal line—insufficient compensating curve. To increase this we usually find that it can be done by changing the position of the second molars alone—making the curvature more abrupt or the arc of a smaller circle. Should we find in the incisive movement of the mandible that there is no incisal contact—that there is molar contact only—then we know that there is too much or too pronounced a compensating curvature; the curvature must be made flatter. This, too, can usually be done by changing the second molars alone.

After an articulation has been tested and corrected in the mouth it is perhaps needless to say that the plates should not be returned to the articulator, for the teeth will not articulate there if alterations were made in the mouth. Should the plates be returned to the articulator, great care must be exercised not to disturb the articulation made in the mouth. Unintentional shifting of the teeth may easily be made when plates are returned to the articulator.

